# Multiscale homogenized constrained mixture model of the bio-chemo-mechanics of soft tissue growth and remodeling

**DOI:** 10.1007/s10237-024-01884-w

**Published:** 2024-10-17

**Authors:** Daniel Paukner, Jay D. Humphrey, Christian J. Cyron

**Affiliations:** 1 Institute for Continuum and Material Mechanics, Hamburg University of Technology, Hamburg, Germany; 2 Institute of Material Systems Modeling, Helmholtz-Zentrum Hereon, Geesthacht, Germany; 3 Department of Biomedical Engineering, Yale University, New Haven, CT USA

**Keywords:** Homeostasis, Constrained mixture, Cell signaling, Growth and remodeling, Soft tissue

## Abstract

Constrained mixture models have successfully simulated many cases of growth and remodeling in soft biological tissues. So far, extensions of these models have been proposed to include either intracellular signaling or chemo-mechanical coupling on the organ-scale. However, no version of constrained mixture models currently exists that includes both aspects. Here, we propose such a version that resolves cellular signal processing by a set of logic-gated ordinary differential equations and captures chemo-mechanical interactions between cells by coupling a reaction-diffusion equation with the equations of nonlinear continuum mechanics. To demonstrate the potential of the model, we present 2 case studies within vascular solid mechanics: (i) the influence of angiotensin II on aortic growth and remodeling and (ii) the effect of communication between endothelial and intramural arterial cells via nitric oxide and endothelin-1.

## Introduction

Most soft tissues consist of a collection of different cell types that communicate with each other and work together to promote tissue homeostasis under normal conditions. According to Adler et al. (2018), nearly all tissues are composed of 4 key cell types: those responsible for the primary function of the tissue plus endothelial cells, fibroblast-like stromal cells, and macrophages. This ensemble forms a remarkably robust system working reliably under different environmental conditions.

To study the response of soft tissue to changes in the mechanical environment on the macroscale, constrained mixture models (Humphrey and Rajagopal 2002) and in particular their homogenized versions (Cyron et al. 2016) have proven very successful. For example, they have been used to study the formation of aortic (Wilson et al. 2013; Mousavi and Avril 2017; Horvat et al. 2019) or cerebral (Baek et al. 2006) aneurysms, vascular adaptation to increased blood pressure (Valentín et al. 2008; Latorre et al. 2019), optimization of tissue-engineered vascular grafts (Szafron et al. 2019), inflammatory effects (Hill et al. 2018; Latorre et al. 2019; Maes et al. 2023), or cardiac growth and remodeling (Gebauer et al. 2023). In both full and homogenized models, the production of new tissue mass is usually governed by phenomenological gain parameters. While this approach allows numerically efficient organ-scale simulations, it does not allow detailed studies of subcellular processes and their effects on the organ-scale.

To study cellular signal processing, it is necessary to model the biochemical reactions within cells. These are usually triggered by signals sensed by receptors on the cell membrane (Alberts et al. 2017). One possibility is to model the reactions in detail, which requires parameters for each involved chemical reaction (Saucerman et al. 2003). This detailed approach, however, requires extensive experimental data to determine the reaction parameters. A simpler alternative is a graph representation of the signaling network (Kraeutler et al. 2010). In this approach, biochemical reactions are replaced by simpler Hill-type functions, resulting in a logic-gated system of ordinary differential equations (ODEs) requiring fewer parameters. Despite its simplicity, this approach has yielded surprisingly accurate results, ranging from cardiac fibroblast signaling (Zeigler et al. 2016) to brain endothelial cells in the context of cerebral pathologies (Gorick et al. 2022). This approach also facilitates studying genetic defects, such as knockdowns or overexpression of specific proteins and pharmacological treatments (Irons and Humphrey 2020).

By combining constrained mixture models and cellular signaling models (Irons et al. 2021), one can create a scale-bridging model that captures how cell signaling affects growth and remodeling (G&R) on the organ-scale and vice versa (Karakaya et al. 2022; van Asten et al. 2022, 2023; Irons et al. 2022). Such scale-bridging models can benefit from the RNA sequencing data that are becoming more and more available. For example, this approach has been used to study effects of exogenous angiotensin II on aortic smooth muscle cells (Irons et al. 2021) and effects of notch signaling in hypertension (van Asten et al. 2023).

Whereas the above studies couple cell signaling and tissue-level constrained mixture models, others focus on chemo-mechanical coupling on the organ-scale (Marino et al. 2017; Gierig et al. 2021). That is, effects of the diffusion of paracrine signals at the tissue level were modeled by coupling reaction-diffusion equations and those for the soft tissue mechanics. Thereby, some have examined interactions of macrophages, smooth muscle cells, and endothelial cells and their effects on the mechanical properties of collagen (Marino et al. 2017); others have examined effects of matrix metalloproteinases (MMPs) and growth factors on the healing process of damaged soft tissue (Gierig et al. 2021). So far, to the authors’ knowledge, no constrained mixture models of G&R include both intracellular signaling and chemo-mechanical coupling on the organ-scale.

In this paper, we introduce the first (homogenized) constrained mixture model that includes both intracellular signaling and chemo-mechanical coupling on the organ-scale for arbitrary geometries. Numerical implementation is based on open-source software packages (deal.ii (Arndt et al. 2021), Trilinos (The Trilinos Project Team 2021), SUNDIALS (Hindmarsh et al. 2005)). By making the model and the associated code available to the community, we aim to facilitate the study of how different aspects of cellular signal processing, such as (dysfunctional) intracellular signaling pathways or cell–cell communication, affect G&R on the organ-scale.

## Mathematical model

### Mechanics

Herein, we use the homogenized constrained mixture theory to describe the mechanics of soft biological tissues undergoing growth (changes in mass) and remodeling (changes in microstructure). Its main equations will be summarized in the following. For more details, the reader is referred to previous articles (Cyron et al. 2016; Braeu et al. 2017; Mousavi et al. 2019; Maes and Famaey 2023).

#### Kinematics

Using the general theory of nonlinear continuum mechanics, soft biological tissue can be modeled as a domain $B_0$ of material points $\varvec{X}$ referred to as reference configuration. A time-dependent deformation maps the domain $B_0$ to its current configuration *B*(*s*) at G&R time *s*. This means that each material point $\varvec{X}$ is mapped to its current position $\varvec{x}(s, \varvec{X})$. Without loss of generality, we assume that the reference configuration coincides with the initial configuration, that is, $B_0 = B(s = 0)$. The so-called deformation gradient at time *s* is

1 $$\begin{aligned} \varvec{F}(s) = \frac{\partial \varvec{x}(s)}{\partial \varvec{X}}. \end{aligned}$$

The determinant of the deformation gradient, $\text{det}(\varvec{F})$, maps differential volume elements *dV* of the reference configuration to differential volume elements *dv* of the current configuration with

2 $$\begin{aligned} dv = \text {det}(\varvec{F}) dV. \end{aligned}$$

**Fig. 1:**
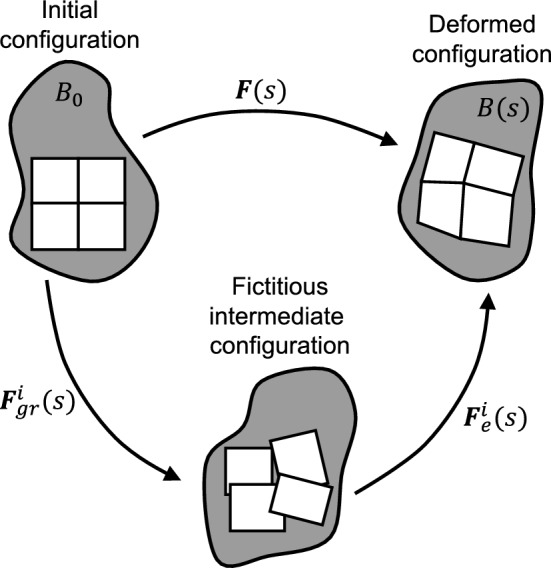
Kinematics of the homogenized constrained mixture model: For each constituent *i*, the deformation gradient $\varvec{F}$ can be split into an inelastic part $\varvec{F}_{gr}^i$ and an elastic part $\varvec{F}_{e}^i$

Although we assume that each volume element is a mixture of *N* structurally significant constituents that are constrained to move together, each constituent has its individual stress-free reference configuration. For each constituent *i*, the deformation gradient $\varvec{F}$ can be split into an inelastic part $\varvec{F}_{gr}^i$ and an elastic part $\varvec{F}_{e}^i$. $\varvec{F}_{gr}^i$ represents the inelastic change of the tissue geometry due to G&R; it maps differential volume elements to a fictitious intermediate configuration that is not necessarily geometrically compatible. That is, neighboring volume elements in the intermediate configuration may overlap, or gaps may arise between them. The volume elements in the intermediate configuration are mapped by $\varvec{F}_{e}^i$ to the current configuration. This happens such that the current configuration is always geometrically compatible and in mechanical equilibrium (Fig. 1).

In the homogenized constrained mixture model, the elastic part of the deformation gradient of a constituent *i* at time *s* is given by

3 $$\begin{aligned} \textbf{F}_e^i(s) = \textbf{F}(s) \textbf{F}_{gr}(s)^{-1} = \textbf{F}(s) \textbf{F}_g(s)^{-1} \textbf{F}_r^i(s)^{-1}. \end{aligned}$$

In this case, $\textbf{F}$ is the deformation gradient of the mixture as a whole, $\textbf{F}_g$ captures the inelastic change of geometry due to growth of all constituents together (change of tissue mass), and $\textbf{F}_r^i$ captures the inelastic part of the deformation gradient resulting from remodeling due to mass turnover of each constituent *i*, where $\textbf{F}_{gr} = \textbf{F}_r^i\textbf{F}_g$. Both $\textbf{F}_g$ and $\textbf{F}^i_r$ have to be defined via evolution equations described below.

#### Elasticity

Since growth and remodeling occur on long time scales, we solve the quasi-static equilibrium equation $div(\varvec{\sigma }) = \textbf{0}$ where $\varvec{\sigma }$ is the Cauchy stress of the mixture. The strain energy of the mixture per unit reference volume is

4 $$\begin{aligned} \Psi = \sum _i \rho _0^i W^i \left( \textbf{C}_e^i \right) . \end{aligned}$$

Here, a superscript $i=e$ refers to elastin, $i=m$ to (circumferential) smooth muscle, and $i = c_0, c_{90}, c_{+\alpha _0}, c_{-\alpha _0}$ to 4 different collagen fiber families with the subscript indicating the angle with respect to a specific direction or axis (collectively summarized as $c_j$). A superscript *c* without an indication of an angle refers to a quantity for collagen as a whole. $W^i$ denotes the strain energy per unit mass of constituent *i*, $\rho ^i_0$ the reference apparent mass density of constituent *i*, and $\textbf{C}_e^i = \textbf{F}_e^{iT} \textbf{F}_e^{i}$ the elastic part of the right Cauchy–Green tensor of constituent *i*. Since inelastic deformations, that is, G&R, do not contribute to the stored energy, the strain energy of the constituents only depends on the elastic part of the deformation.

The main constituents of the tissues considered herein are an isotropic elastin matrix, reinforced by several families of quasi-one-dimensional fibers of collagen and smooth muscle. The elastin matrix is modeled by the strain energy (Braeu et al. 2017; Maes and Famaey 2023)

5 $$\begin{aligned} W^e = \frac{\mu ^e}{2} \left( \bar{I}^e_1 - 3\right) + \frac{\kappa }{2} \left( J_e^e - 1\right) ^2 \end{aligned}$$

where $\mu ^e$ and $\kappa$ are material parameters, $\bar{I}^e_1$ is the trace of the isochoric elastic Cauchy–Green tensor of elastin $\bar{\textbf{C}}_e^e$ (including prestretch), and $J_e^e$ is the determinant of the elastic part of the deformation gradient of elastin, $\textbf{F}_e^e$. Note that $\bar{\textbf{C}}_e^e = \left( J_e^e \right) ^{-2/3} \textbf{F}_e^{eT} \textbf{F}_e^{e}$ (Holzapfel 2000). The quasi-one-dimensional fibers reinforcing the elastin matrix are modeled using Fung-type constitutive equations

6 $$\begin{aligned} W^\alpha = \frac{c_1^\alpha }{4c_2^\alpha } \left[ \exp { \left( c_2^\alpha \left( I_{4e}^\alpha - 1\right) ^2 \right) } - 1\right] \end{aligned}$$

with the superscript $\alpha$ referring to fiber constituents (i.e., $\alpha = m, c_j$), $I_{4e}^\alpha = \left( \textbf{a}_{gr}^\alpha \right) ^T \textbf{C}_e^\alpha \textbf{a}_{gr}^\alpha$ the fourth pseudo-invariant of the elastic Cauchy–Green tensor of constituent $\alpha$ and $\textbf{a}_{gr}^\alpha$ its fiber orientation in the inelastically deformed intermediate configuration (Braeu et al. 2017; Gebauer et al. 2023). Note that $I_{4e}^\alpha = \left( \lambda _e^\alpha \right) ^2$ with the elastic fiber stretch $\lambda _e^\alpha$.

In certain cases, an additional active stress contribution of the smooth muscle cells may be considered and added in Eq.  (4) as part of their strain energy. In this case, the stress of smooth muscle is the sum of the passive and active contributions. This is based on the assumption that active and passive contributions act in parallel with the contractile units embedded in a passive matrix (Murtada et al. 2010). Following Wilson et al. (2012), we model this contribution as

7 $$\begin{aligned} W^m_{act} = \frac{\sigma _{max}}{\rho _0(0)} y_{con}(s) \left[ \lambda _{act} + \frac{\left( \lambda _{max} - \lambda _{act} \right) ^3}{\left( \lambda _{max} - \lambda _0 \right) ^2}\right] . \end{aligned}$$

Here, $\sigma _{max}$ is the maximal active stress where the factor $y_{con}(s) \in [0, 1]$ modulates that stress according to intracellular signaling in a way described in Sect.  2.2. The stretches $\lambda _{max}$ and $\lambda _0$ are the stretches at which force generation is maximal and minimal, respectively. The active smooth muscle stretch in the fiber direction $\lambda _{act}$ is modeled as $\lambda _{act} = {\lambda }/{\hat{\lambda }_{act}}$ where $\lambda$ is the total stretch in the fiber direction and $\hat{\lambda }_{act}$ represents the (evolving) reference configuration for active smooth muscle. We assume that this reference configuration evolves due to fast muscle remodeling such that $\hat{\lambda }_{act} = \lambda$ (Wilson et al. 2012). Hence, $\lambda _{act} = {\lambda }/{\hat{\lambda }_{act}} = 1$ at all times *s* in the quasi-static equilibrium but ${\partial \lambda _{act}}/{\partial \lambda } = {1}/{\lambda }$ (Braeu et al. 2017).

#### Growth

Different possibilities exist for defining the growth tensor $\textbf{F}_g$. For simplicity, we choose an anisotropic growth tensor (Braeu et al. 2017) used in several studies (Mousavi and Avril 2017; Maes and Famaey 2023). In this case, the growth tensor in equation (3) can be written as

8 $$\begin{aligned} \textbf{F}_g = \frac{\rho _0(s)}{\rho _0(0)} \textbf{a}_g \otimes \textbf{a}_g + \left( \textbf{I} - \textbf{a}_g \otimes \textbf{a}_g \right) \end{aligned}$$

with $\textbf{a}_g$ the unit growth direction vector in the reference configuration, $\rho _0(s) = \sum _i \rho _0^i(s)$, and $J_g = \text {det}(\textbf{F}_g) = \rho _0(s)/\rho _0(0)$. For ease of comparison with previous studies, we chose to use transversely isotropic growth, noting that other ways of defining the growth tensor can be implemented in the model (Braeu et al. 2019).

#### Remodeling

The evolution equation for $\textbf{F}_r^i$ in equation (3) is detailed in (Cyron et al. 2016) and (Maes and Famaey 2023); see these articles for details. Briefly, constituents are subject to continuous mass turnover where extant tissue mass is degraded and new mass is added. This process adapts the stress-free configuration of a constituent over time in a manner that is, in many respects, similar to inelastic deformations in viscoelasticity. Assuming new mass is added with a preferred stress $\varvec{\sigma }_h^i$, this can effectively be captured for constituent *i* by an evolution equation

9 $$\begin{aligned} \left[ \frac{ \dot{\rho }^i_{{0}}}{\rho ^i_{0}} + \frac{1}{T^i} \right] \left[ \varvec{\sigma }^i - \varvec{\sigma }_h^i \right] = \left[ \frac{\partial \varvec{\sigma }^i}{\partial \textbf{F}_e^i} :\left( \textbf{F}_e^i \textbf{L}_r^i \right) \right] _{\varvec{F},\varvec{F}_g =const.} \end{aligned}$$

where $\textbf{L}_r^i = \dot{\textbf{F}}_r \left( \textbf{F}_r \right) ^{-1}$ represents the remodeling velocity gradient, $\dot{\rho }^i_{0}$ the rate of change of the referential mass density $\rho ^i_0$, $T^i$ the degradation time constant (Cyron et al. 2016; Maes and Famaey 2023; Gebauer et al. 2023; Braeu et al. 2017), and $\varvec{\sigma}_h^i$ the assumed homeostatic target value for the Cauchy stress experienced by constituent *i*. We assume that only the fiber constituents representing collagen and smooth muscle ($\alpha = m, c_j$) are subject to G&R. Further assuming incompressible remodeling of the fibers, $\textbf{F}_r^{\alpha }$ can be written as (Cyron et al. 2016)

10 $$\begin{aligned} \textbf{F}_r^{\alpha } = \lambda _r^{\alpha } \textbf{a}_0^{\alpha } \otimes \textbf{a}_0^{\alpha } + \frac{1}{\sqrt{\lambda _r^{\alpha }}} \left( \textbf{I} - \textbf{a}_0^{\alpha } \otimes \textbf{a}_0^{\alpha } \right) \end{aligned}$$

with $\textbf{a}_0^{\alpha }$ the fiber orientation in the reference configuration, and $\lambda _r^{\alpha }$ the inelastic remodeling stretch in the fiber direction, the only unknown. Hence, equation (9) can be simplified to a scalar evolution equation for $\dot{\lambda }_r^{\alpha }$ (see Appendix 1 in (Cyron et al. 2016) or (Maes and Famaey 2023) for a derivation)

11 $$\begin{aligned} \dot{\lambda }_r^{\alpha } = \left( \frac{ \dot{\rho }^{\alpha }_{0}}{\rho ^{\alpha }_{0}} + \frac{1}{T^{\alpha }}\right) \frac{ \lambda ^{\alpha }_r}{2 I_{4e}^{\alpha }} \left[ \frac{\partial {\sigma }^{\alpha }}{\partial I_{4e}^{\alpha }} \right] ^{-1} \left( {\sigma }^{\alpha } - {\sigma }_h^{\alpha } \right) . \end{aligned}$$

This evolution equation can be integrated using standard time integration schemes; we use a forward Euler scheme. Following (Gebauer et al. 2023), the initial remodeling stretch is set to

12 $$\begin{aligned} \lambda _r^{\alpha }(0) = \frac{1}{\lambda _h^{\alpha }} \end{aligned}$$

where $\lambda _h^{\alpha }$ represents the (homeostatic) deposition stretch of constituent ${\alpha }$. This ensures a homeostatic initial state for constituents subject to G&R.

#### Initial configuration

The reference (and also initial) configuration with $\textbf{F} = \textbf{I}$ is typically not load-free; we assume that it is a homeostatic state. That is, the constituents subject to G&R (collagen ($\alpha = c_j$) and smooth muscle ($\alpha = m$)) are in their respective homeostatic state with an initial elastic part of the deformation gradient

13 $$\begin{aligned} \textbf{F}^{\alpha }_e(s=0) = \lambda ^{\alpha }_h \textbf{a}_{0}^{\alpha } \otimes \textbf{a}_{0}^{\alpha } + \frac{1}{\sqrt{\lambda ^{\alpha }_h}} \left( \textbf{I} - \textbf{a}_{0}^{\alpha } \otimes \textbf{a}_{0}^{\alpha } \right) , \end{aligned}$$

with $\lambda ^{\alpha }_h$ the homeostatic stretch of collagen or smooth muscle, and $\textbf{a}_{0}^{\alpha }$ the orientation of the respective fiber family in the reference configuration (Cyron et al. 2016; Braeu et al. 2019; Maes et al. 2019).

We determine the initial elastic stretch of elastin such that the constrained mixture as a whole is in mechanical equilibrium at $\textbf{F} = \textbf{I}$ for a given external loading (e.g., the blood pressure $p_0$ in a vessel) and that smooth muscle and collagen are in their respective homeostatic state (Bellini et al. 2014). An iterative approach is adopted to this end as detailed before (Mousavi and Avril 2017; Famaey et al. 2018; Maes et al. 2019). As a starting point for the iterations, an axial prestretch of elastin $\lambda _z^e$ (estimated, e.g., from experimental data) is prescribed, and the initial elastin prestretch tensor is constructed assuming incompressibility (Famaey et al. 2018)

14 $$\begin{aligned} \textbf{F}^e_{e,k=0}(s=0) = \begin{bmatrix} \frac{1}{\sqrt{\lambda _z^e}} &{} 0 &{} 0\\ 0 &{} \frac{1}{\sqrt{\lambda _z^e}} &{} 0\\ 0 &{} 0 &{} \lambda _z^e \end{bmatrix}. \end{aligned}$$

At iteration step *k*, a mechanical equilibrium problem is solved based on an assumed elastic prestretch $\textbf{F}^e_{e,k}(s=0)$ of elastin and yields for the constrained mixture as a whole the deformation gradient $\textbf{F}_k$ compared to the desired initial state. The objective of the iterations is to achieve $\textbf{F}_k = \textbf{I}$, with *k* the iteration step, which indicates that the elastic prestretch of elastin has been chosen such that mechanical equilibrium is satisfied directly in the desired initial configuration. To achieve $\textbf{F}_k = \textbf{I}$, we compute for iteration step $k+1$ for elastin the assumed elastic prestretch $\textbf{F}^e_{e,k+1}(s=0) = \textbf{F}_k \textbf{F}^e_{e,k}(s=0)$ and continue such iterations until we have found a $\textbf{F}^e_{e,k+1}(s=0)$ which yields within a certain tolerance $\textbf{F}_{k+1} = \textbf{I}$ when solving the associated mechanical equilibrium problem. To facilitate the solution, external forces, predefined deposition stretches of the fiber constituents, and the axial deposition stretch of elastin are applied gradually in the first few iterations.

### Intracellular signaling

As mentioned in Sect. 1, different approaches exist to model the intracellular signaling network of cells. We focus on the approach introduced by Kraeutler et al. (2010), which, despite its relative simplicity, has produced excellent results (Zeigler et al. 2016; Irons and Humphrey 2020; Gorick et al. 2022). This model uses a logic-based representation of the chemical reactions of cellular signal processing. Each chemical species is represented by a node $y_i$ in a directed graph. Under normal conditions, the value of each node ranges in the continuous interval [0, 1] with 0 representing an inactive state and 1 a fully active state. However, diseases or pharmacological treatments might change the maximal activity level of a node to some different upper bound $y_{i,max}$, which will be discussed at the end of this section. In a graph of *N* nodes, the change of activation of node $y_i$ is given by the following (nonlinear) ODE,

15 $$\begin{aligned} \frac{dy_i}{ds} = \frac{1}{\tau _{i}} f_i(y_1, \dots , y_N) y_{i,max} - y_i \end{aligned}$$

where $\tau _{i}$ is a time constant (determining the reaction speed). To understand this model, consider the simplest case where node $y_i$ depends only on one other node $y_j$. In this scenario, we distinguish 2 cases. First, $y_j$ is assumed to have a positive (activating) effect on $y_i$, and $f_i(y_1, \dots , y_N)$ is assumed to reduce to

16 $$\begin{aligned} f_i(y_j) = f^{+}_{i}(y_j):= W_{ij} \frac{B_{ij} y_j^{n_{ij}}}{(B_{ij} - 1)^{n_{ij}} + y_j^{n_{ij}}}. \end{aligned}$$

**Fig. 2:**
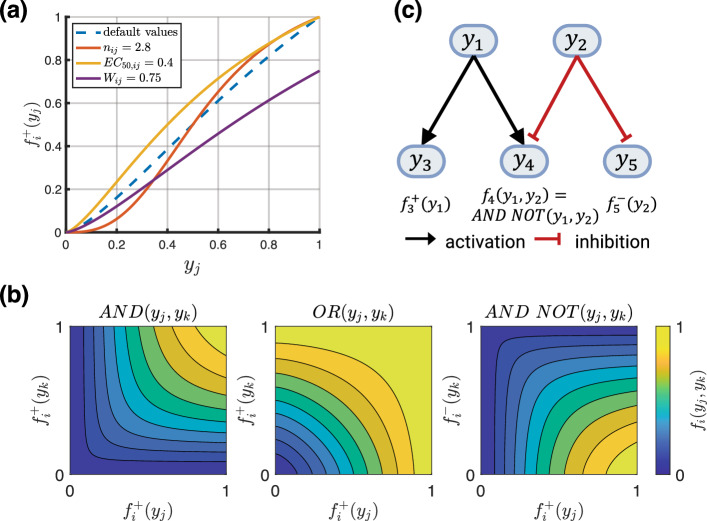
**a** Dashed blue line shows activation function with default parameters $W_{ij} = 1$, $EC_{50,ij}=0.5$, $n_{ij}=1.4$, solid lines show variations of a single parameter (see legend) from the default case and its effect on the activation function. **b** Contour plots of logical operators described by the Eqs.  (20) through (22). **c** A simple example of a signaling network with 2 input nodes ($y_1$, $y_2$) and 3 output nodes ($y_3$, $y_4$, $y_5$)

This function is illustrated in Fig. 2a. Second, if $y_j$ has a negative (inhibitory) effect on $y_i$, $f_i(y_1, \dots , y_N)$ is assumed to be (Kraeutler et al. 2010; Khalilimeybodi et al. 2020)

17 $$\begin{aligned} f_i(y_j)&= f^{-}_{i}(y_j) := 1 - f^{+}_{i}(y_j) \nonumber \\&= 1 - W_{ij} \frac{B_{ij} y_j^{n_{ij}}}{(B_{ij} - 1)^{n_{ij}} + y_j^{n_{ij}}} . \end{aligned}$$

Activating functions of the type $f^{+}_{i}$ and inhibitory functions of the type $f^{-}_{i}$ similarly form the main building blocks of a signaling network in more complex scenarios. They represent normalized Hill functions, with $n_{ij}$ the Hill coefficient, $W_{ij}$ a reaction weight, and the constant $B_{ij}$ enforcing the constraints

18 $$\begin{aligned} f^{+}_{i}(y_j = 0)&= 0, \nonumber \\ f^{+}_{i}(y_j = 1)&= W_{ij}, \nonumber \\ f^{+}_{i}(y_j = EC_{50,ij})&= 0.5 W_{ij} \end{aligned}$$

where $EC_{50,ij}$ is the value at which the activation function is half-maximal, i.e., $0.5 W_{ij}$. Based on these constraints, $B_{ij}$ is

19 $$\begin{aligned} B_{ij}&= \frac{\left( EC_{50,ij}\right) ^{n_{ij}} - 1}{2 \left( EC_{50,ij}\right) ^{n_{ij}} - 1} \end{aligned}$$

with $\left( EC_{50,ij} \right) ^{n_{ij}} < 0.5$ to ensure $B_{ij}$ remains positive. If a node $y_i$ depends on 2 nodes $y_j$ and $y_k$, their combined effect is modeled by the logical operations ‘AND,’ ‘OR,’ and ‘AND NOT’ (Irons et al. 2022). In the first of these 3 cases, $y_i$ is activated if both $y_j$ and $y_k$ are active and we have

20 $$\begin{aligned} f_{i}(y_j, y_k) = AND(y_j, y_k):= \frac{2 f^{+}_{i}(y_j) f^{+}_{i}(y_k)}{ f^{+}_{i}(y_j) + f^{+}_{i}(y_k)}. \end{aligned}$$

In the second case, $y_i$ is activated if $y_j$ or $y_k$ are active and we have

21 $$\begin{aligned} f_{i}(y_j, y_k)&= OR(y_i, y_j) \nonumber \\&:= f^{+}_{i}(y_j) + f^{+}_{i}(y_k) - f^{+}_{i}(y_j) f^{+}_{i}(y_k). \end{aligned}$$

In the third case, $y_i$ is activated if $y_j$ is active and $y_k$ is inactive, and we have

22 $$\begin{aligned} f_{i}(y_j, y_k)&= AND \ NOT(y_i, y_j) \nonumber \\&:= \frac{2 f^{+}_{i}(y_j) f^{-}_{i}(y_k)}{f^{+}_{i}(y_j) + f^{-}_{i}(y_k)} \nonumber \\&= \frac{2 f^{+}_{i}(y_j) \left( 1 - f^{+}_{i}(y_k)\right) }{f^{+}_{i}(y_j) + \left( 1 - f^{+}_{i}(y_k)\right) }. \end{aligned}$$

These logical operators are illustrated in Fig. 2b. Note that Eqs.  (20) and (22) deviate slightly from the original definitions (Kraeutler et al. 2010) and use a modification (Khalilimeybodi et al. 2020; Irons et al. 2022) to ensure proper scaling. This avoids issues where the classical ‘AND’ definition, $f^{+}_{i}(y_j) f^{+}_{i}(y_k)$, would lead to outputs that are too small if not scaled by $2 / \left( f^{+}_{i}(y_j) + f^{+}_{i}(y_k)\right)$ (similar for ‘AND NOT’). If a node $y_i$ depends on several other nodes, their effect on $y_i$ can be modeled by a recursive combination of the logical operators in Eqs.  (20) through (22).

Based on the above model, the dynamics of the network are governed by a system of nonlinear ODEs. A simple example with 5 nodes is illustrated in Fig. 2c. The inputs could represent, e.g., the stress of the smooth muscle fibers (node $y_1$) and the nitric oxide concentration in the tissue (node $y_2$). The outputs could represent smooth muscle proliferation (node $y_3$), collagen production (node $y_4$), and the active contraction of smooth muscle (node $y_5$). By changing $y_{i,max}$, it is possible to simulate overexpression ($y_{i,max} > 1$) or knockdown ($y_{i,max} < 1$) of a species that could result from a pathogenic variant or a pharmacological treatment.

### Continuum-scale biochemistry

#### Reaction-diffusion equations

**Fig. 3:**
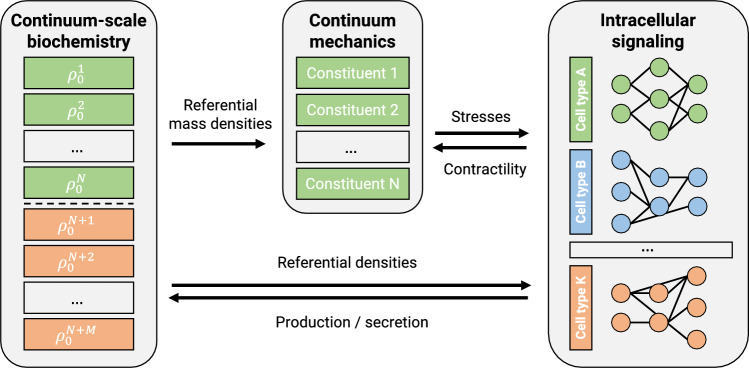
Schematic coupling of mechanics, intracellular signaling, and reaction-diffusion problem (continuum-scale biochemistry). Only the first *N* species in the reaction-diffusion problem are assumed to be structurally significant (elastin, collagen, smooth muscle), whereas the last *M* species in the concentration vector are assumed to be structurally insignificant but to matter as an environment for the intracellular signaling processes

One way cells communicate is by secreting signaling molecules that diffuse through the surrounding tissue and are sensed by neighboring cells (paracrine signaling) or the cell itself (autocrine signaling). We model such processes on the continuum scale by a coupled system of reaction-diffusion equations

23 $$\begin{aligned} \frac{\partial \rho ^k_0}{\partial s} = D^k_0 \nabla ^2 \rho ^k_0 + r^k_0(\rho ^1_0,..., \rho ^{N+M}_0) + s^k_0(s) \end{aligned}$$

with superscript *k* denoting the *k*th species tracked by the model in the extracellular space ($k = 1,..., N+M$). Here, $\rho ^k_0$ is the density in the reference configuration of species *k*, *N* is the number of structurally significant species (such as elastin, collagen, smooth muscle), and *M* is the number of relevant biochemical species, for example, signaling molecules secreted by the cells that are not structurally significant (see Fig. 3). $D^k_0$ is the (constant) diffusion coefficient of species *k*, $r^k_0$ represents possible reactions between species, and $s^k_0$ is a potentially time-dependent source term. The source term can, for example, represent the exogenous addition of certain molecules or potential endocrine signals.

In the present coupled chemo-mechanical model (see below), the tracked species include the $N=3$ structural constituents of the soft tissue (elastin, collagen, smooth muscle) and potentially the concentrations of *M* biochemical species, such that $k = e, c, m,..., N+M$. For the 3 mechanically relevant constituents in the constrained mixture, we assume

24 $$\begin{aligned} D^c_0&= 0, \nonumber \\ r^c_0&= \frac{1}{T^c} \frac{\rho _0^c(0)}{\rho _0^m(0)} \rho _0^m (s) (1 + \Delta \psi ^c(s)) - \frac{1}{T^c} \rho _0^c (s), \nonumber \\ s^c_0&= 0, \end{aligned}$$

25 $$\begin{aligned} D^m_0&= 0,\nonumber \\ r^m_0&= \frac{1}{T^m} \rho _0^m (s) (1 + \Delta \psi ^m(s)) - \frac{1}{T^m} \rho _0^m (s), s^m_0&= 0. \end{aligned}$$

For elastin, we assume no deposition, degradation, or diffusion, that is, $s^e_0=0$, $r^e_0=0$, $D^e_0=0$. By contrast, we assume that collagen production depends on the intramural cell density since more cells, all producing collagen at a basal rate, leads to more collagen production. Here, the factor $\frac{\rho _0^c(0)}{\rho _0^m(0)}$ ensures a homeostatic state at G&R time $s=0$. The $T^i$ are degradation time constants and the $\Delta \psi ^i(s)$ are normalized deviations of the relevant signaling network output of node $y_{i}$ from its baseline level $y_{i0}$ with (Irons et al. 2021)

26 $$\begin{aligned} \Delta \psi ^i(s) = \frac{y_{i}(s) - y_{i0}}{y_{i0}}. \end{aligned}$$

Following Irons et al. (2021, 2022), we assume a constant relative distribution of the different collagen fiber families, which leads to

27 $$\begin{aligned} \frac{\dot{\rho }_0^{c_j}}{\rho _0^{c_j}} = \frac{\dot{\rho }_0^{c}}{\rho _0^{c}}. \end{aligned}$$

For simplicity, we assume that rates of collagen degradation and cell apoptosis are constant and $D^c_0 = D^m_0 = 0$.

Note that the purely mechanical homogenized constrained mixture model can be obtained by setting $D^{\alpha }_0 = 0$, $s^{\alpha }_0=0$ and

28 $$\begin{aligned} r^{\alpha }_0 = \rho ^{\alpha }_0(s) k^{\alpha } \frac{\sigma ^{\alpha }(s) - \sigma ^{\alpha }_h}{\sigma ^{\alpha }_h},\quad \alpha = m, c_j \end{aligned}$$

where $k^{\alpha }$ is a rate parameter, $\sigma ^{\alpha }(s)$ the current stress of fiber constituent $\alpha$, and $\sigma ^{\alpha }_h$ its homeostatic stress (Braeu et al. 2017; Gebauer et al. 2023). In this case, equation (27) need not hold.

#### Endothelium as a boundary condition

Endothelial cells can play important roles in sensing mechanical stimuli as well as in cell–cell communication. To include these effects, an endothelial cell layer with its own signaling network can be added to the model. For example, when modeling a blood vessel by a tube-like geometry, the endothelial cells line the inner surface. Using the current (inner) radius of the vessel, the mean wall shear stress can be computed based on

29 $$\begin{aligned} \tau _w = \frac{4 \mu Q}{\pi a^3}, \end{aligned}$$

assuming a fully developed laminar flow of a Newtonian fluid. Here, $\mu$ is the fluid viscosity, *Q* the flow rate, and *a* the current (inner) radius. Changes in wall shear stress can then serve as an input to the endothelial signaling network and trigger a response. For example, by releasing signaling molecules, which diffuse through the attached volume, the endothelial cells can communicate with intramural cells by providing potential inputs to their signaling networks. Note that the endothelial cells are assumed not to be structurally significant but instead form a (dynamic) Neumann-type boundary condition for the continuum-scale diffusion problem described in the previous section.

## Implementation

### Coupling

The equations in Sect. 2 related to mechanics, intracellular signaling, and continuum-scale biochemistry are coupled as illustrated in Fig. 3. For example, nodes in the intracellular signaling model determine mass production rates in the continuum-scale biochemistry model; these production rates govern the G&R that drives mechanical deformation; these deformations result in mechanical stress, which alters inputs for the intracellular signaling model.

Hence, the above equations must be solved as a coupled system. A geometry, for example, a tube representing a blood vessel, is discretized using the finite element method (FEM) to solve both mechanical and continuum-scale biochemical reaction-diffusion problems. The intracellular signaling is computed at a representative selection of material points, that is, at each Gauss point in each finite element. To solve time-dependent problems, we discretize time into a finite number of times $s^i$ with $i=1,2,3,\ldots$. At each time $s^i$, we solve the coupled problem via the following sequence of steps:

1. FEM solution of mechanical problem based on system state at previous time $s^{i-1}$,
2. Solution of ODE system for intracellular signaling (to steady state) at each Gauss point in the FEM discretization based on the mechanical state computed in the previous step (1) and mass concentrations from $s^{i-1}$,
3. FEM solution of reaction-diffusion problem with mechanical state from step (1) and intracellular signaling state from step (2).

This staggered solution scheme allows a flexible combination of submodules. For example, in certain cases, only 2 of the 3 domains of the problem may actually be solved (e.g., only mechanics and diffusion or only mechanics and intracellular signaling).

### Signaling network initialization

To determine normalized changes $\Delta \psi ^i(s)$ of pathway output $y_i$ in (26), its baseline pathway output $y_{i0}$ is needed. This value can be determined by fixing all input nodes to a baseline value and simulating the dynamics of the network until a steady state at all output nodes has been reached.

### Scaling of inputs for intracellular signaling

**Fig. 4:**
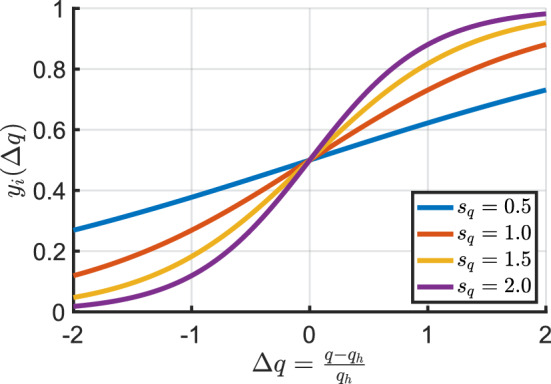
Influence of sensitivity $s_q$ on scaling behavior with $y_{i0} = 0.5$

To use a continuum-level quantity *q* (e.g., smooth muscle stress) as an input to a node $y_i$ in the intracellular signaling network, it has to be scaled to a suitable range. A simple way is to use a sigmoidal function

30 $$\begin{aligned} y_i(\Delta q) = \frac{1}{1 + e^{\beta - s_{q} \cdot \Delta q}}, \quad \text {with } \Delta q = \frac{q - q_h}{q_h} \end{aligned}$$

where $s_{q}$ determines the sensitivity to a perturbation from homeostatic, $\Delta q$ represents the normalized deviation of *q* from its baseline (e.g., homeostatic) value, $q_h$, and $\beta$ shifts the sigmoidal function horizontally such that $y_i(\Delta q = 0) = y_{i0}$ where $y_{i0}$ is the baseline input at time $s = 0$. Figure 4 shows the influence of varying sensitivities on the scaling behavior.

### Solvers

#### Mechanics

The nonlinear mechanical equilibrium problem was solved with a standard Newton solver from the Trilinos NOX package (The Trilinos Project Team 2021) (relative and absolute tolerances set to $10^{-12}$). The linearized system was solved with a direct solver since all the problems studied were relatively small. The code was fully parallelized, supporting iterative linear solvers in case of larger problems.

#### Intracellular signaling

The system of ODEs modeling the intracellular signaling was solved using the SUNDIALS ARKode package (Hindmarsh et al. 2005; Gardner et al. 2022; Reynolds et al. 2023) with an explicit Runge–Kutta method of order 5 with relative and absolute tolerances of $10^{-10}$. Because ARKode is a general-purpose ODE solver, there is no restriction on using a logic-based model of intracellular signaling.

#### Continuum-scale biochemistry

To solve the reaction-diffusion problem modeling continuum-scale biochemistry, the SUNDIALS ARKode package was used with an implicit Runge–Kutta method of order 3 in combination with a GMRES solver and an algebraic-multigrid (AMG) preconditioner. The relative and absolute tolerances of the time integration algorithm were set to $10^{-7}$ and $10^{-8}$, respectively.

## Numerical examples

### Validation of mechanical model

To validate the implementation of the (uncoupled) homogenized constrained mixture model of G&R (Eq. (28)), we recreated the test cases presented in (Maes and Famaey 2023). See Appendix A.1 for details.

### Growth and remodeling of murine aorta

**Table 1:** Parameters for coupled finite element simulations of growth and remodeling of a mouse descending thoracic aorta

*Geometry and material parameters*		
Mixture mass density	$\rho _0$	$1050\ kg/m^3$
Initial mass fractions	$\phi ^e$	0.34
	$\phi ^m$	0.33
	$\phi ^{c_0}$, $\phi ^{c_{90}}$, $\phi ^{c_{\pm \alpha _0}}$	0.01848, 0.02211, 0.1447
Collagen fiber orientation	$\alpha _0$	$29.91^{\circ }$
Initial inner radius	*A*	$0.647\ mm$
Initial wall thickness	*H*	$0.04\ mm$
Initial length	*L*	$0.04\ mm$
Elastin parameter	$\mu ^e$	$89.71\ kPa$
Elastin bulk modulus	$\kappa$	$10 \mu ^e$
Collagen properties	$c^c_1,\ c^c_2$	$234.9\ kPa,\ 4.08$
SMC properties	$c^m_1,\ c^m_2$	$261.4\ kPa,\ 0.24$
Elastin axial deposition stretch	$\lambda ^e_z$	1.62
Collagen deposition stretch	$\lambda ^{c}_h$	1.25
SMC deposition stretch	$\lambda ^{m}_h$	1.2
Mean survival times	$T^m,\ T^c$	$70\ days,\ 70\ days$
Initial blood pressure	$p_0$	$14.0\ kPa$
Time step size	$\Delta s$	$7\ days$
*Continuum-scale biochemistry parameters*		
SMC diffusion coefficient	$D^m_0$	0
Collagen diffusion coefficient	$D^c_0$	0
*Cell signaling parameters*		
SMC sensitivity to stress	$s_{\sigma }$	0.5
Reaction time constant	$\tau$	1.0
Reaction edge weight	*W*	1.0
Half maximal activation	$EC_{50}$	0.55
Hill parameter	*n*	1.25
Maximal node activation	$y_{max}$	1.0
Initial input values	$y_{WSS,0}$	0.5
	$y_{integrins,0}$	0.2
	$y_{SACs,0}$	0.2
	$y_{stress,0}$	0.2
	$y_{AngII,0}$	{0.0, 0.1}

Geometry and material parameters are based on (Irons et al. 2022; Latorre and Humphrey 2020) and represent radially averaged values for a single-layered G&R model. Cell signaling parameters are based on (Irons and Humphrey 2020).

In the following section, we use the coupled model to study the response of a mouse descending thoracic aorta to mild to moderate increases in pressure. To this end, we coupled the homogenized constrained mixture model to the signaling pathways introduced by Irons and Humphrey (2020) and further used and refined in (Irons et al. 2021, 2022). The network consisted of 50 nodes and 82 reactions and represents the behavior of synthetic intramural cells (SMCs and fibroblasts) as one might obtain from bulk qPCR or Western blotting. The network has 5 inputs (Angiotensin II (AngII), smooth muscle stress, flow-induced wall shear stress (WSS), integrins, stretch-activated channels (SACs)) and 4 outputs (Collagen-I, Collagen-III, cell proliferation, actomyosin activity). The outputs Collagen-I and Collagen-III were combined into one output for fibrillar collagen production by taking their average, noting that this is a simplification as collagen fiber formation is highly complex and the contribution of other matrix constituents is omitted (Kadler et al. 2008; Liu et al. 1997). For the network, we used the default parameters from (Irons and Humphrey 2020) unless noted otherwise, see Table 1. Inputs to the network were scaled using the sigmoidal function in equation (30). In Sect.  4.2.1, the only variable input to the signaling network is the smooth muscle stress, while in Sect.  4.2.2, the inputs are the smooth muscle stress, the nitric oxide, and endothelin-1 concentration.

The geometry and material parameters are based on (Irons et al. 2022; Latorre and Humphrey 2020) and represent a radially averaged single-layered arterial wall. Assuming axisymmetry, the artery was modeled as a cylindrical quarter segment similar to Sect. A.1.2 with original inner radius *A*, thickness *H*, and length *L* (Table 1). Axial displacements were fixed at both ends, and the growth direction defined as radial. The geometry was discretized using 216 quadratic finite elements: 2 in radial and axial directions, and 54 elements in the circumferential direction. The choice of 2 quadratic elements in the radial direction was motivated by a convergence study (Latorre and Humphrey 2020) that used 4 linear elements in radial direction. This was confirmed by a mesh convergence study. The geometry was prestressed using the algorithm described in Sect.  2.1.5 with an *in vivo* pressure of $p_0 = 14.0\ kPa$ until the average nodal displacement (normalized by the reference wall thickness) was less than $10^{-6}$ and a homeostatic state was (nearly) reached.

#### Cell signaling network - influence of AngII

**Fig. 5:**
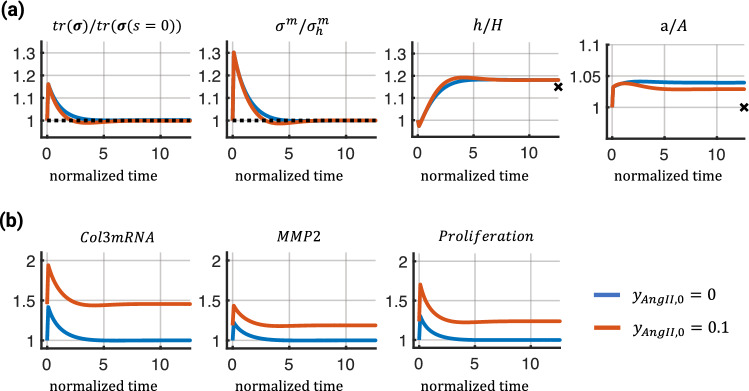
Mechanical and network responses of the coupled finite element model in response to a 15% pressure increase under 2 different levels of exogenous AngII over normalized time ($s/T^m$). Top (**a**) shows, from left to right, the normalized trace of the mixture-level Cauchy stress, normalized Cauchy stress of the smooth muscle fibers, normalized wall thickness, and normalized inner radius. The dashed lines represent baseline values and markers represent ideal adaptations based on theoretical considerations (see text). Bottom (**b)** shows fold changes in activation levels of 3 network nodes with respect to the baseline ($y_{AngII,0} = 0$)

In the first study, we compared how the presence of AngII via modification of the baseline network input $y_{AngII,0}$ influences arterial G&R when subjected to a sustained sudden increase in pressure of 15%. To quantify tissue adaption over time in response to the pressure perturbation, we computed the normalized trace of the tissue-level Cauchy stress and the normalized Cauchy stress of the smooth muscle cells at the midpoint of the vessel. Additionally, we show normalized wall thickness and inner radius. In this example, we assumed that flow rate *Q* remains constant at its initial value $Q_0$, yielding a flow rate ratio $\varepsilon = Q/Q_0 = 1$ and a pressure ratio $\gamma = p/p_0$, where *p* is the blood pressure after the pressure increase. It is known (Humphrey 2008) that blood vessels tend to regulate their inner radius in accordance with the flow rate (to maintain a certain wall shear stress) and their wall thickness in accordance with the inner pressure (to maintain a certain hoop stress). The markers at the right end of the graphs in the third and fourth columns represent adaptations that would be expected from these theoretical considerations. To maintain the initial wall shear stress, the (current) inner radius, *a*, should adapt to the limit $\varepsilon ^{1/3} A$; to maintain a certain hoop stress, the (current) wall thickness, *h*, should adapt to the limit $\gamma \varepsilon ^{1/3} H$ (Humphrey 2008).

As shown in Fig. 5a, exogenous AngII accelerated the adaptive response leading to a slight undershoot in the trace of the mixture-level Cauchy stress and the SMC stress before recovering baseline values (first 2 graphs). The adapted wall thickness was similar in both cases and close to the ideal adaptation based on the theoretical considerations (third graph). However, the adapted inner radii were different; the simulation with AngII showed a smaller radius (fourth graph). These results qualitatively aligned with experimental observations (Wu et al. 2014; Ruddy et al. 2009) that AngII increases collagen turnover, leading to accelerated remodeling. This is, for example, indicated by increased activation of the network nodes representing Col3mRNA and MMP2 (Fig. 5b). However, note that the simulations should be viewed as a proof of concept as the model still missed important aspects such as wall shear stress sensing, and the parameters were not calibrated to specific experimentally observed results.

#### Endothelial cell–intramural cell communication

**Fig. 6:**
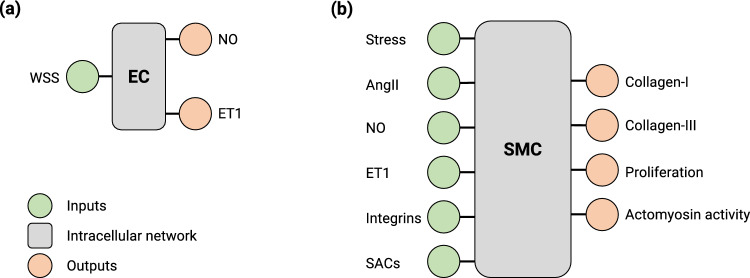
Schematic illustration of the inputs and outputs of the endothelial cell signaling network (**a**) and the intramural cell signaling network (**b**). NO production by ECs increases with increases in WSS; ET1 production increases with decreases in WSS

To ensure an adaptive response of the tissue, different cells and cell types need to communicate with each other, for example, through signaling molecules. One such example of cell–cell communication can be observed between endothelial cells (ECs) and intramural cells such as smooth muscle cells and fibroblasts. ECs in a blood vessel respond to changes in wall shear stress (WSS), $\tau _w$, by changing their production of nitric oxide (NO) and endothelin-1 (ET1) (Sriram et al. 2016), which alter smooth muscle contractility (Hong et al. 2011) and collagen production (Hershey et al. 2012). Even though the interaction between the 2 cell types is much more complex, involving other signaling molecules and growth factors, we reduced their interaction to depend only on nitric oxide and endothelin-1. For the following simulations, we also included active SMC stresses, as NO and ET1 are potent regulators of active smooth muscle tone. The parameter regulating active cell contraction $y_{con}(s)$ used in Eq. (7) is computed via a sigmoidal scaling function (Eq.  (30)) with sensitivity 1.0, $\Delta \psi ^{act}(s)$ is the normalized change of the output node in the signaling network regulating actomyosin activity, and $y_{con,0} = 0.5$.

In addition to cells within the vessel wall, we included an EC layer on the inner surface as described in Sect.  2.3.2. We used the same intracellular signaling pathway as in Sect.  4.2.1 but extracted the part that represents endothelial cell behavior and assigned it to the ECs, while the remaining network was assigned to the intramural cells, which now have NO and ET1 inputs instead of wall shear stress (Fig. 6).

The ECs respond to changes in wall shear stress (WSS) by producing NO and ET1, which then diffuse through the vessel wall with diffusion coefficients $D^{NO}_0$ and $D^{ET1}_0$, but are consumed or degrade with mean survival times $T^{NO}$ and $T^{ET1}$ assuming first-order kinetics (Vaughn et al. 1998; Liu et al. 2008). The parameter values are based on previous experimentally determined values (Malinski et al. 1993) or theoretical studies (Vaughn et al. 1998; Liu et al. 2008; Kavdia and Popel 2006) and listed in Table 2, including references where available. Compared to NO, data on ET1 are scarce. Hence, we used a generic value for the diffusion coefficient of a peptide, and the baseline production rate was assumed to be of a similar order of magnitude as NO.

**Table 2:** Additional parameters for the coupled finite element simulations including endothelial cell–intramural cell communication

*Material parameters*			
SMC active contribution	$\sigma _{max}$	$170\ kPa$	Irons et al. (2022)
	$\lambda _{max}$	1.1	Irons et al. (2022)
	$\lambda _0$	0.4	Irons et al. (2022)
*Continuum-scale biochemistry parameters*			
Nitric oxide diffusion coefficient	$D^{NO}_0$	$3300\ \mu m^2/s$	Malinski et al. (1993); Vaughn et al. (1998)
Nitric oxide mean survival time	$T^{NO}$	15*s*	Malinski et al. (1993); Vaughn et al. (1998)
Nitric oxide production rate	$\dot{Q}_0^{NO}$	$5.3 \cdot 10^{-14}\frac{\mu mol}{\mu m^2 s}$	Vaughn et al. (1998)
Endothelin-1 diffusion coefficient	$D^{ET1}_0$	$300\ \mu m^2/s$	–
Endothelin-1 mean survival time	$T^{ET1}$	120*s*	Saleh et al. (2016)
Endothelin-1 production rate	$\dot{Q}_0^{ET1}$	$1.0 \cdot 10^{-14}\frac{\mu mol}{\mu m^2 s}$	see text
Fraction diffusing into tissue	$\eta$	0.2	see text
Outflux fraction	$\xi$	0.9	see text
*Cell signaling parameters*			
EC sensitivity to wall shear stress	$s_{\tau }$	0.5	–
SMC sensitivity to nitric oxide	$s_{NO}$	0.75	–
SMC sensitivity to endothelin-1	$s_{ET1}$	0.75	–
SMC sensitivity to changes in actomyosin activity	$s_{con}$	1.0	–

Active smooth muscle parameters are based on (Irons et al. 2022)

One challenge in this scenario was the boundary conditions. Similar to (Vaughn et al. 1998; Kavdia and Popel 2006), we relate the NO and ET1 production rates $\dot{Q}_0^{NO}$ and $\dot{Q}_0^{ET1}$ to the influx of these 2 quantities and prescribed corresponding boundary conditions,

31 $$\begin{aligned} \dot{Q}_0^{NO} (1 + \Delta \psi ^{NO}(s))&= D^{NO}_0 \frac{\partial [NO]_{lumen}}{\partial r} \nonumber \\&- D^{NO}_0 \frac{\partial [NO]_{tissue}}{\partial r} \end{aligned}$$

32 $$\begin{aligned} \dot{Q}_0^{ET1} (1 + \Delta \psi ^{ET1}(s))&= D^{ET1}_0 \frac{\partial [ET1]_{lumen}}{\partial r} \nonumber \\&- D^{ET1}_0 \frac{\partial [ET1]_{tissue}}{\partial r}. \end{aligned}$$

Here, $\Delta \psi ^{NO}(s)$ and $\Delta \psi ^{ET1}(s)$ are outputs from the EC signaling network regulating NO and ET1 production, while $\frac{\partial [NO]}{\partial r}$ and $\frac{\partial [ET1]}{\partial r}$ represent concentration gradients in the radial direction (into the lumen or into the tissue). Some of the NO and ET1 produced by the ECs will be released into the lumen and transported away by convection, whereas some parts will be released into the tissue and transported via diffusion. We estimate the fraction of NO and ET1 that is released into the tissue to be $\eta = 0.2$ (Vaughn et al. 1998; Liu et al. 2008; Zhang and Edwards 2006). This resulted in the following boundary conditions on the inner surface of the vessel,

33 $$\begin{aligned} - D^{NO}_0 \frac{\partial [NO]_{tissue}}{\partial r}&= \eta \dot{Q}_0^{NO} (1 + \Delta \psi ^{NO}(s)) \end{aligned}$$

34 $$\begin{aligned} - D^{ET1}_0 \frac{\partial [ET1]_{tissue}}{\partial r}&= \eta \dot{Q}_0^{ET1} (1 + \Delta \psi ^{ET1}(s)). \end{aligned}$$

Additionally, a second boundary condition is needed at the outer surface of the vessel wall. Because the outer surface of the vessel is not impermeable to NO and ET1 and the vessel is surrounded by perivascular tissue, an outflux boundary condition was prescribed. Previous studies (Vaughn et al. 1998; Liu et al. 2008; Zhang and Edwards 2006) found that the concentration profile of NO is nearly linear over short distances comparable to the wall thickness of the studied vessel. Hence, we assumed that the outflux is proportional to the influx with a proportionality factor $\xi$. Here, we assumed that $\xi = 0.9$, which resulted in a nearly linear concentration profile of NO and ET1 and concentrations on the order of 100-300 *nM* (Chen et al. 2008).

**Fig. 7:**
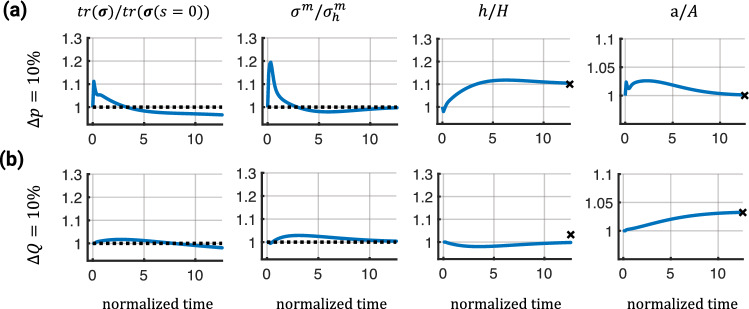
Evolution of selected quantities over normalized time ($s/T^m$) in response to a 10% pressure increase (**a**) or a 10% flow increase (**b**). Dashed lines represent baseline values and markers indicate theoretical ideal adaptations for the different cases

To analyze the influence of EC–transmural cell communication on tissue adaptation, we first applied a sudden 10% pressure increase that was sustained over time. The additional stimulus through NO and ET1, mediated by wall shear stress sensing of the endothelial cells, led to an almost ideal adaptation in inner radius and wall thickness (Fig. 7a). After a slight undershoot, the stress of the SMCs slowly returned back toward the baseline value while a slight offset in the trace of the tissue-level Cauchy stress remained.

With the addition of the EC layer and the NO-mediated cell–cell communication, the model could now also account for changes in blood flow rate. To study the response of the vessel to a change in (blood) flow, we increased the flow rate *Q* by 10% ($\varepsilon = Q/Q_0 = 1.1$) while keeping the blood pressure constant ($\gamma = p/p_0 = 1$). As illustrated in Fig. 7b, the vessel dilated to reduce the wall shear stress close to the value $\varepsilon ^{1/3} A \approx 1.03 A$ with $\varepsilon = Q/Q_0 = 1.1$. The stresses slowly returned toward their baseline values with a slight offset in the trace of the tissue-level Cauchy stress. After a slight decrease, the thickness of the vessel remained unchanged compared to the initial thickness, different from the idealized adaptation limit $\gamma \varepsilon ^{1/3} H$.

### Instabilities for high sensitivities

When analyzing the results from the previous Sects.  4.2.1 and 4.2.2, we found that simulations can exhibit instabilities at longer simulation times (normalized time > 20). These instabilities resulted in a continuous deviation of the stresses from their baseline values, ultimately leading to divergence of the simulations. To further probe this behavior, we replaced the complex intracellular signaling pathway with a highly simplified model consisting of only 3 nodes: one input node (smooth muscle stress) that activated 2 outputs (cell proliferation and collagen production). For this network, $\tau = 1$, $W=1$, $EC_{50}=0.5$, $n=1.4$, and $y_{max}=1$. Smooth muscle stress as an input for the intracellular signaling network was scaled to the range [0, 1] by a sigmoidal function with an adjustable sensitivity as described in Eq. (30). Five sensitivities, $s_{\sigma }$, ranging from 0.25 to 1.25, were tested for a 5%, 10%, and 15% pressure increase.

**Fig. 8:**
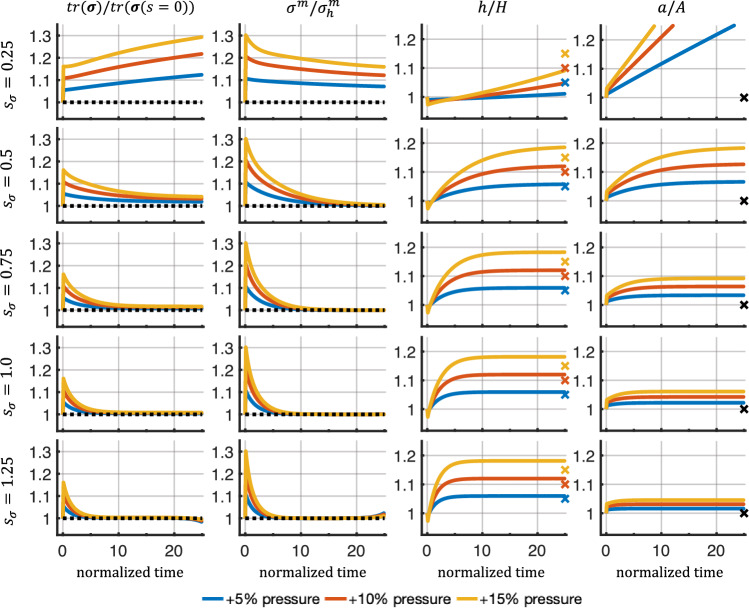
Evolution of selected quantities over normalized time ($s/T^m$) for varying sensitivities in response to different step increases in pressure (5%, 10%, 15%). Dashed lines represent baseline values, and markers indicate ideal adaptations based on applied pressure perturbation

**Fig. 11:**
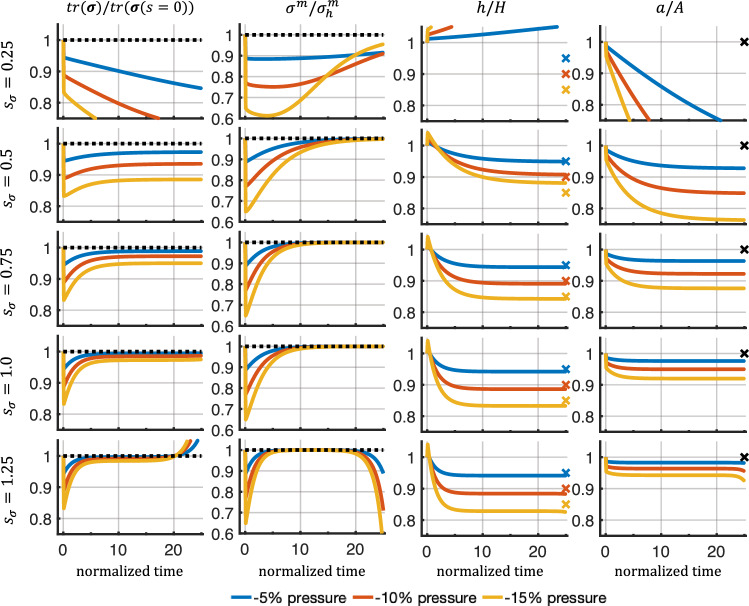
Evolution of selected quantities over normalized time ($s/T^m$) for varying sensitivities in response to step decreases in pressure (-5%, -10%, -15%). Notably, for low sensitivities, we observed immediate instability. For very high sensitivities, we again observed long-term instabilities. Both are qualitatively similar to what was observed for pressure increases

Figure 8 shows that low sensitivities (e.g., $s_{\sigma }=0.25$) led to unbound growth and continuous distension of the vessel. This is in agreement with a prior observation (Braeu et al. 2017) for low rate parameters, $k^i$, using a homogenized constrained mixture model without intracellular signaling. In fact, too low sensitivities $s_{\sigma }$ led to mechanobiological instabilities (Cyron and Humphrey 2014; Cyron et al. 2014). By contrast, higher sensitivities led to a regime of mechanobiological stability, marked by the convergence of the vessel geometry to a new stable mechanobiological equilibrium state. Higher sensitivities decreased the offset in the trace of the tissue-level Cauchy stress compared to its initial value. This is in agreement with the concept of mechanobiological adaptivity (Cyron and Humphrey 2014; Cyron et al. 2014). The response in case of corresponding pressure decreases is shown in Fig. 11 in Appendix B.1.

**Fig. 12:**
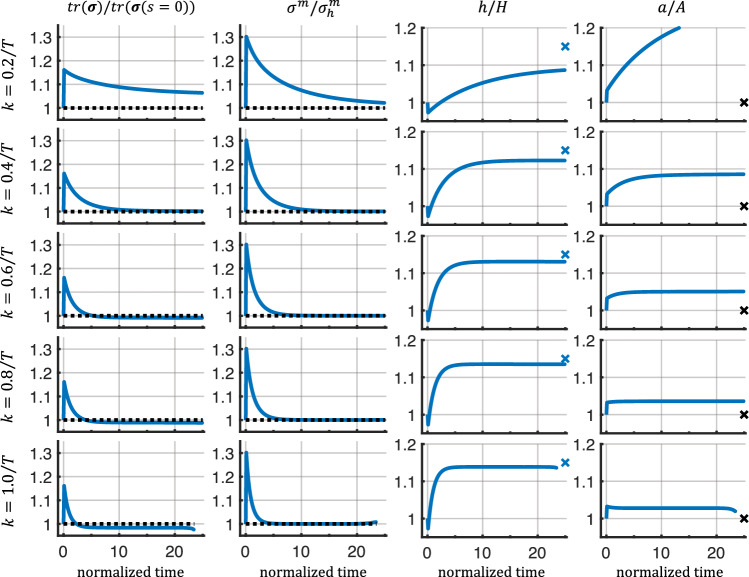
Evolution of selected quantities over normalized time ($s/T^m$) in a purely mechanical homogenized constrained mixture model following a pressure increase of 15% with rate parameters $k = {k^m} = k^c$ ranging from 0.2/*T* to 1.0/*T* with $T = T^m = T^c$ the mean survival time of smooth muscle and collagen, respectively

**Fig. 13:**
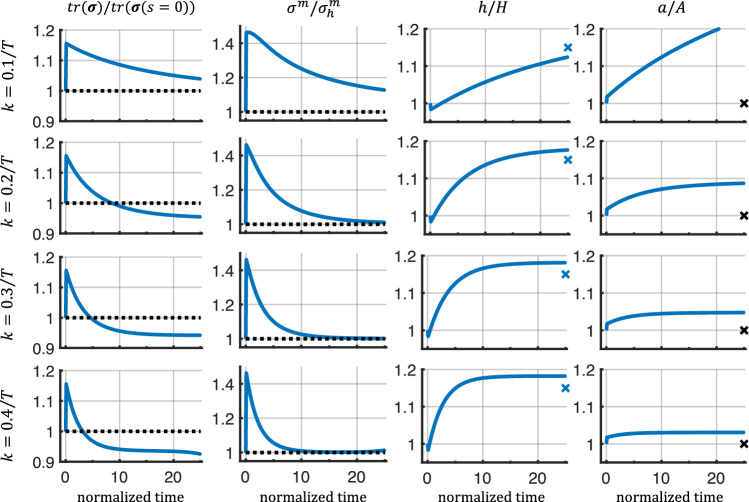
Evolution of selected quantities over normalized time ($s/T^m$) in a purely mechanical homogenized constrained mixture model following a pressure increase of 15% with the parameters from Braeu et al. (2017) (Table 4), using rate parameters $k = {k^m} = k^c$ ranging from 0.1/*T* to 0.4/*T* with $T = T^m = T^c$ the mean survival time of smooth muscle and collagen, respectively

For the highest sensitivity ($s_{\sigma }=1.25$), instabilities in the stresses were observed toward the end of the simulation. Interestingly, these instabilities seem independent of the magnitude of the applied perturbations and were not immediately visible in geometric quantities (thickness and inner radius). To ensure that the instabilities were not introduced through the coupling of the different submodules, we tested the uncoupled homogenized constrained mixture model with the same geometry, material parameters, and a pressure increase of 15%. We found similar instabilities when increasing the rate parameters, $k^i$, to high values, which is comparable to increasing the sensitivity $s_{\sigma }$ (Fig. 12 in Appendix B.2). Further testing using the uncoupled homogenized constrained mixture model with the geometry and material parameters described in Braeu et al. (2017) again revealed instabilities for high rate parameters when subjected to a pressure increase of 15% (Fig. 13 in Appendix B.2).

**Fig. 14:**
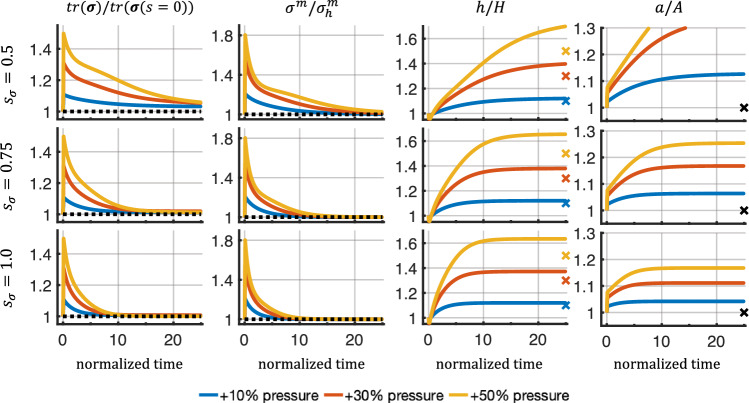
Evolution of selected quantities over normalized time ($s/T^m$) in a coupled multiphysical model with pressure increases of 10%, 30%, and 50%. The model parameters are listed in Table 1

Furthermore, we tested if the sensitivities leading to stable adaptations ($s_{\sigma } = \{0.5, 0.75, 1.0\}$) would show instabilities under pressure perturbations of 30% and 50% (Fig. 14 in Appendix B.3). No stress instabilities were observed in these cases. However, a sensitivity of 0.5 seemed insufficient to achieve a stable state within the observed time period. Further tests of reducing the time step size, gradual application of the pressure perturbations, or using stricter tolerances did not remove the instabilities.

## Discussion

### Intracellular signaling

Adding intracellular signaling networks to the homogenized constrained mixture model to replace phenomenological parameters for mass production with quantities rooted in intracellular signaling allows a more detailed study of how specific intracellular processes influence tissue homeostasis. While the example presented in Sect.  4.2.1 should be understood as a proof of principle, it showed how signaling networks could be used to study how G&R is affected by a different biochemical environment, in this case, the presence of AngII. In these cases, it can be difficult to know how certain chemicals influence phenomenological parameters for collagen production and cell proliferation a priori. Furthermore, the complex intracellular signaling networks can also exhibit internal feedback loops, which can lead to a more dynamic adaptation process, such as the undershoot of the stresses or the reduction in radius observed in Fig. 5.

In addition to the influence of exogenous factors, it becomes possible to study how specific defects in intracellular signaling, such as a genetic defect, change the behavior on the organ-scale. Notably, some defects in intracellular signaling may have little effect on the organ-scale due to compensatory mechanisms in the signaling networks. Generally, biological signaling networks benefit from this kind of robustness as their surrounding is inherently noisy and they have to operate under various conditions. This may explain why a unique parametrization of the (logic-gated) signaling networks is challenging to obtain but also might not be necessary to promote a stable adaptation (Irons et al. 2022).

Moreover, the intracellular signaling pathways can be created with the amount of detail necessary for the studied problem. For example, with the highly simplified signaling networks used in Sect.  4.3, we observed that a range of (stress) sensitivities can promote a stable adaptive response. Similar results were obtained with purely mechanical homogenized constrained mixture models, where a specific range of rate parameters led to stable adaptations (Appendix B.2). However, the coupled model allows a more straightforward interpretation of the physiological meaning and origin of the parameters.

### Effects of cell–cell communication

It is known that different cell types collectively contribute to tissue homeostasis. In fact, 4 cell types appear to be sufficient to form a minimal tissue unit: (I) the cell type performing the core function (for example, smooth muscle cells in the aorta, hepatocytes in the liver, etc.), (II) endothelial cells, (III) fibroblast-like stromal cells, and (IV) tissue-resident macrophages. According to Adler et al. (2018), the last 3 cell types are nearly universal to all tissues and mainly support the primary cell type in their function.

In this work, we included endothelial cells in addition to the intramural cells. The combination of both cell types communicating with each other via NO and ET1 produced different results (Fig. 7a) compared to a model where only intramural cells were included (Fig. 5). The additional influence of the NO and ET1, regulated by the wall shear stress sensed by the ECs, allowed the vessel to adjust its inner radius toward ideal values. This underlines one of the many important functions of the endothelium. It also allowed the study of tissue adaptation in response to changes in flow (Fig. 7b), which, to the best of our knowledge, was so far not included in 3-dimensional simulations using the homogenized constrained mixture model.

Overall, coupled models have the potential to study different combinations of cell types forming basic tissue units. For example, including macrophages could help to elucidate their role in promoting tissue homeostasis (Zhou et al. 2018). In particular, studying dysfunctional communication between different cell types and the resulting effects on tissue adaptation could improve our understanding of when tissue can adapt and under which circumstances it cannot. This might help to identify potential pharmacological targets or treatments to improve clinical outcomes.

The flexibility of the model to include several cell types offers great potential but can also add considerable complexity. Ultimately, though, it is a trade-off between the amount of detail to include, and the resulting complexity to still generate interpretable and actionable results or hypotheses, which can then be tested experimentally.

### Long-term instabilities

Long-term instabilities might arise when high rate parameters, $k^i$, or high sensitivities, $s_{\sigma }$, are used in combination with curved geometries such as the cylinder wall studied herein. Interestingly, this was independent of the magnitude of the applied pressure perturbation. We found that adding diffusion of the cells (i.e., $D_0^m > 0$ in Eq. (25)), which could represent cell migration, was able to stabilize the system for all times considered and even larger values of $k^i$ and $s_{\sigma }$ (not shown). However, this lacks a biological foundation, at least in the case of elastic arteries, where migration of SMCs is constrained by the elastic laminae, and fibroblasts primarily remain in the adventitia. Nevertheless, these results might indicate that the homogenized constrained mixture model is not able to homogenize the transmural stress gradients and maintain them toward the end of the adaptation process.

The question remains as to what exactly causes these instabilities potentially resulting from persistent transmural stress gradients. The instabilities might be inherent to the homogenized constrained mixture model, caused by basic assumptions, or of a purely numerical nature. For example, Taber and Humphrey (2001) showed that radially varying material parameters help to homogenize the transmural stresses. Moreover, Azeloglu et al. (2008) showed that a gradient in the proteoglycan concentration also provides a mechanism by which transmural stresses can be homogenized, and cells might modify proteoglycan synthesis and degradation in response to altered stresses. These observations are not included in current implementations. Additionally, we used a radially averaged, single-layered approach to model the arterial wall. However, the arterial wall consists of 3 distinct layers, the endothelium, media, and adventitia, the latter 2 bearing the majority of the load. This bi-layered structure can significantly influence transmural stresses. For example, Bellini et al. (2014) showed that different material properties in the medial and adventitial layer can protect the media from excessive stresses when pressure increases above normal as the adventitia carries more load at elevated pressure levels.

As evidenced by the different possibilities listed above, transmural stress gradients are important and complex, and different mechanisms have been identified that influence their distribution. More work is needed to understand this better and examine what exactly causes the long-term instabilities in the homogenized constrained mixture model when large rate parameters or sensitivities are used and how this influences transmural stress distributions.

## Conclusion and outlook

In this paper, we proposed the first homogenized constrained mixture model of soft tissue growth and remodeling that couples mechanics, continuum-scale biochemistry, and intracellular signaling. Our model allows one to replace phenomenological growth parameters of purely mechanical homogenized constrained mixture models with models of specific intracellular processes. This allows one to relate a behavior on the organ-scale to specific intracellular processes. Moreover, it facilitates physiological interpretations of the simulations. Importantly, the continuum-scale biochemistry module in our framework also allows us to capture long-range communication between cells, e.g., the communication between endothelial cells and transmural cells via nitric oxide and endothelin-1.

The computational cost of our coupled multiphysical model was around 3–5 times higher than that of a purely mechanical homogenized constrained mixture model of soft tissue G&R. The majority of the additional cost can be attributed to the implicit time integration scheme used in the diffusion submodule and solving the intracellular signaling networks. The former could potentially be improved by using different time integration schemes. The computational cost of solving the intracellular signaling strongly depends on their size and complexity. However, the additional level of detail will always incur an additional computational cost.

In the future, the model could be used to study specific aspects of cell–cell communication or the effect of genetic defects on growth and remodeling. Moreover, it provides insights into how biochemical processes on the cell-level can translate into macroscopic changes on the tissue level. While the presented results are promising, more research is needed to make the coupled model applicable to real-world applications. Independent of the coupling to intracellular signaling networks, the instabilities in the homogenized constrained mixture model occurring during longer simulations with large rate parameters or stress sensitivities need to be addressed to find their cause.

## Figures and Tables

**Fig. 9:**
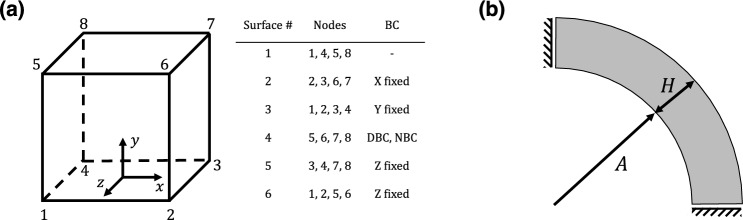
Test cases used for verification based on Maes and Famaey (2023). **a** Schematic drawing of a unit cube and the corresponding boundary conditions (BC) for each surface. DBC - Dirichlet boundary condition, NBC - Neumann boundary condition. **b** Schematic of arterial growth and remodeling test case

**Fig. 10:**
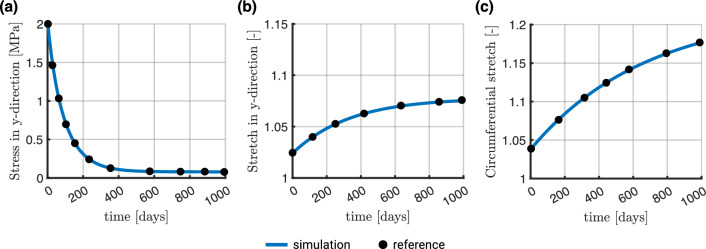
Results of our simulations (continuous line) vs. reference data from Maes and Famaey (2023) (black markers). **a** Cube subjected to displacement boundary condition. **b** Cube subjected to (Cauchy) traction boundary condition. **c** Circumferential stretch at the inner surface of the cylinder wall in response to a 50% pressure increase. The reference data were obtained using WebPlotDigitizer (Rohatgi 2022)

## Data Availability

The code used for this article is available at https://github.com/pauknerd/multiscale_mixture.

## Appendix A

### A.1 Validation of mechanical model

The implementation of the (uncoupled) homogenized constrained mixture model of G&R (Eq. (28)) is validated based on test cases presented in (Maes and Famaey 2023): a unit cube with either a displacement or traction boundary condition and a cylindrical tube as a simplified model of arterial adaptations to a pressure increase, see Fig. 9. The material parameters for both cases are largely identical and summarized in Table 3. Note that while the elastin content is higher than *in vivo*, the material parameters suggested by Maes and Famaey (2023) fall into physiological relevant ranges and are comparable to parameters used in previous studies such as (Wilson et al. 2012) who fitted experimental data from (Vande Geest et al. 2004) or (Latorre and Humphrey 2018) who fitted experimental data from (Bersi et al. 2016).

**Table 3:** Geometry and material parameters for the test cases based on Maes and Famaey (2023). DBC - Dirichlet boundary condition

*Geometry - Cube*		
Edge length	L	$1\ mm$
Homeostatic Cauchy stress	$\sigma _0$	$0.1\ MPa$
*Geometry - Artery*		
Initial inner radius	A	$5\ mm$
Initial wall thickness	H	$1.3\ mm$
Initial length	L	$1.3\ mm$
Initial pressure	$p_0$	$0.01\ MPa$
*Material parameters*		
Initial mass fractions	$\phi ^e$	0.8
	$\phi ^{c_0}$, $\phi ^{c_{90}}$, $\phi ^{c_{\pm \alpha _0}}$	0.05
Collagen fiber orientation	$\alpha _0$	$22.5^{\circ }$
Elastin parameter	$\mu ^e$	$0.07625\ MPa$
Elastin bulk modulus	$\kappa$	$0.7625\ MPa$
Collagen properties	$c^c_1, c^c_2$	$1.156\ MPa,\ 1.23$
Elastin axial deposition stretch	$\lambda ^e_z$	1.0 (cube with DBC), 1.2
Collagen deposition stretch	$\lambda ^{c}_h$	1.0 (cube with DBC), 1.1
Collagen rate parameter	$k^{c}$	0.1/$T^c$
Mean survival time	$T^c$	$101\ days$
Time step size	$\Delta s$	$10\ days$

#### A.1.1 Unit cube

In the first test case, a unit cube (Fig. 9a) was discretized with a single finite element. The cube consisted of a slightly compressible elastin matrix and 4 families of collagen fibers in the *yz*-plane, including angles of $0^\circ$, $90^\circ$, $+22.5^\circ$, and $-22.5^\circ$ relative to the *y*-axis, respectively. The boundary conditions on the surfaces are as follows: Surface 1 is free, surface 2 is fixed in *x*-direction, surface 3 is fixed in *y*-direction, surface 5 is fixed in *z*-direction, and surface 6 is fixed in *z*-direction. The displacement or stress boundary condition is applied in *y*-direction to surface 4. The growth direction is defined as the *x*-direction.

*Dirichlet boundary condition* In the first test, a displacement of 0.5 *mm* in the *y*-direction was applied to the upper surface of the cube (surface 4), corresponding to a stretch of 1.5. In this case, no prestressing for the initial configuration was applied since the deposition stretches were assumed to be $\lambda ^c_h = \lambda ^e_z = 1.0$. The obtained results compared well to ones from Fig. 2(I) in (Maes and Famaey 2023), see Fig. 10a.

*Neumann boundary condition* In the second test, a Cauchy traction was applied to the upper surface of the cube (surface 4). Since the deposition stretches of collagen and elastin were $\lambda ^c_h = 1.1$ and $\lambda ^e_z = 1.2$, respectively, the iterative prestress algorithm described in Sect.  2.1.5 was used to determine the final prestretch of elastin. To this end, a homeostatic Cauchy stress, $\sigma _0$, of $0.1\ MPa$ was applied in *y*-direction to surface 4, and the prestress algorithm was run for 20 iterations (equivalent to the procedure in (Maes and Famaey 2023)). In the second step, the stress was increased by $20\%$ to $0.12\ MPa$, and the material was allowed to remodel for 100 steps, equivalent to 1000 days. The obtained results compared well with ones from Fig. 2(II) in (Maes and Famaey 2023), see Fig. 10b.

#### A.1.2 Arterial wall remodeling

Next, consider an idealized model of an artery with a simple cylindrical geometry. The inner radius was $A = 5$ mm, the wall thickness $H = 1.3$ mm, and the length $L = 1.3$ mm in the reference configuration. Again, 4 families of collagen fibers were included with angles $0^\circ$, $90^\circ$, $+22.5^\circ$, and $-22.5^\circ$ relative to the circumferential direction. Due to axisymmetry, the geometry was reduced to a quarter cylinder (Fig. 9b) and discretized with 448 linear (hexahedral) finite elements, with 4 elements in radial and axial directions and 28 in the circumferential direction. The growth direction is defined as the radial direction. Material parameters are listed in Table 3. To obtain a prestressed configuration in equilibrium, the iterative prestress algorithm described in Sect. 2.1.5 was used with an *in vivo* pressure of 10 kPa for 20 iterations. Once this configuration was obtained, a $50\%$ pressure increase to 15 kPa (in the current configuration) was applied, and the artery was allowed to grow and remodel over 100 time steps with a time step size of 10 days. Figure 10c depicts the circumferential stretch evolution on the cylinder’s inner surface and compares the result to the one presented in (Maes and Famaey 2023) (Fig. 4(I)).

## Appendix B

### B.1 Response to decreased pressure

We studied how the model discussed in Sect.  4.3 responded to a decrease (rather than an increase) in internal pressure in the cylinder representing a blood vessel. Specifically, we studied pressure decreases of 5%, 10%, and 15%. Similar to the cases of pressure increases, a low sensitivity could not restore a new homeostatic state, while high sensitivities led to instabilities toward the end of the simulation.

### B.2 Instabilities of purely mechanical model for large rate parameters

To examine possible reasons for the long-term instabilities observed in Sect. 4.3 for high sensitivities, we simulated comparable scenarios but with a purely mechanical homogenized constrained mixture model (based on Eq. (28)) using the geometry and material parameters listed in Table 1 and in Table 4. Figures 12 and 13 reveal that also in the case of a purely mechanical homogenized constrained mixture model, long-term instabilities arise for high rate parameters, which are comparable to high sensitivities in the model used in Sect. 4.3. This suggests that the long-term instabilities observed in Sect.  4.3 are not specific for the coupled model presented herein but are rather inherent already to the mechanical homogenized constrained mixture model (likely only in 3-dimensional geometries because they have never been reported in quasi-2-dimensional models using, e.g., membrane finite elements).

**Table 4:** Geometry and material parameters for the simple model aorta used in Braeu et al. (2017)

Mixture mass density	$\rho _0$	$1050\ kg/m^3$
Initial mass fractions	$\phi ^e$	0.23
	$\phi ^m$	0.15
	$\phi ^{c_0}$, $\phi ^{c_{90}}$, $\phi ^{c_{\pm \alpha _0}}$	0.062, 0.062, 0.248
Collagen fiber orientation	$\alpha _0$	$45^{\circ }$
Initial inner radius	*A*	$10\ mm$
Initial wall thickness	*H*	$1.41\ mm$
Initial length	*L*	$1.41\ mm$
Elastin parameter	$\mu ^e$	$72\ J/kg$
Elastin bulk modulus	$\kappa$	$10 \mu ^e$
Collagen properties	$c^c_1,\ c^c_2$	$568\ J/kg,\ 11.2$
SMC properties	$c^m_1,\ c^m_2$	$7.6\ J/kg,\ 0.8$
Elastin axial deposition stretch	$g^e_z$	1.25
Collagen deposition stretch	$\lambda ^{c}$	1.062
SMC deposition stretch	$\lambda ^{m}$	1.1
Mean survival times	$T^c,\ T^m$	$101\ days,\ 101\ days$
Initial blood pressure	$p_0$	$14.0\ kPa$
Time step size	$\Delta s$	$10.1\ days$

### B.3 Response to higher step increases in pressure

We repeated the simulations presented in Sect. 4.3 with higher pressure increases of 30% and 50% for the previously stable (stress) sensitivities of 0.5, 0.75, and 1.0. Figure 14 shows that no long-term instabilities arise for high sensitivities (though for the sensitivity 0.5 and a pressure increase by 30% and 50%, a stable state is not reached within the observed period). Our results support the hypothesis that the magnitude of the pressure perturbation is not the decisive factor for the long-term instabilities observed in Sect. 4.3. It should be noted, of course, that *in vivo* responses to large increases in pressure are expected to elicit an inflammatory response Latorre et al. (2021), not modeled here.
